# Assessing the Impact of Street-View Greenery on Fear of Neighborhood Crime in Guangzhou, China

**DOI:** 10.3390/ijerph18010311

**Published:** 2021-01-04

**Authors:** Fengrui Jing, Lin Liu, Suhong Zhou, Jiangyu Song, Linsen Wang, Hanlin Zhou, Yiwen Wang, Ruofei Ma

**Affiliations:** 1 School of Geography and Planning, Sun Yat-sen University, Guangzhou 510275, China; jingfr@mail2.sysu.edu.cn (F.J.); eeszsh@mail.sysu.edu.cn (S.Z.); songjy7@mail2.sysu.edu.cn (J.S.); wangls3@mail2.sysu.edu.cn (L.W.); marf5@mail2.sysu.edu.cn (R.M.); 2 Guangdong Provincial Engineering Research Center for Public Security and Disaster, Guangzhou 510275, China; 3 Department of Geography, University of Cincinnati, Cincinnati, OH 45221-0091, USA; zhouhl@mail.uc.edu; 4 Center of GeoInformatics for Public Security, School of Geography and Remote Sensing, Guangzhou University, Guangzhou 510006, China; 5 School of Architecture and Urban Planning, Nanjing University, Nanjing 210093, China; dg20360010@smail.nju.edu.cn

**Keywords:** fear of crime, street-view greenery, neighborhood incivilities, social integration, China

## Abstract

Previous literature has examined the relationship between the amount of green space and perceived safety in urban areas, but little is known about the effect of street-view neighborhood greenery on perceived neighborhood safety. Using a deep learning approach, we derived greenery from a massive set of street view images in central Guangzhou. We further tested the relationships and mechanisms between street-view greenery and fear of crime in the neighborhood. Results demonstrated that a higher level of neighborhood street-view greenery was associated with a lower fear of crime, and its relationship was mediated by perceived physical incivilities. While increasing street greenery of the micro-environment may reduce fear of crime, this paper also suggests that social factors should be considered when designing ameliorative programs.

## 1. Introduction

The relationship between fear and vegetated areas has been examined in the literature. In the studies conducted on urban parks, densely wooded areas have consistently been related to fear [1,2]. However, well-maintained or high-visibility vegetation is associated with a lower level of fear [3,4]. Recently, there has been a rising interest in studying the relationship between the amount of urban green space and perceived neighborhood safety [5,6,7].

The urban green space data based on satellite images are widely used by scholars [8,9]. However, the downward-facing satellite represents a bird-eye-view, and cannot accurately reflect people’s perceived green space from the ground-level perspective. In recent years, the Street View Index (SVI) at eye-level [10,11] has been widely used. Eye-level street greenery can more accurately reflect exposures to green space, including small-sized and vertical natural elements (e.g., trees along a street, small plants, and grasses) [11].

Compared with well-known methods, using street-view greenery to examine the relationship between urban greenery and fear of crime has two obvious advantages. First, street-view greenery detects small green elements that might not be detectable from the sky. Second, it captures the actual exposure of individuals to greenery. Individuals’ perception of victimization risk is found to be related to the micro-environments in their routine activity spaces. People do not usually have a bird’s-eye view of their surroundings. Therefore, remote sensing images and the derived data do not represent what people can see on the ground. In contrast, street-level views are far more realistic.

As the most populous country, China has experienced unprecedented rapid urbanization in recent decades, which reduces the opportunities for urban residents to be exposed to the natural outdoor environment [12]. Thus, clarifying the underlying linkage between residential street-view greenery and fear of crime will not only fill the knowledge gap about the Chinese context but also guide policymakers on how to improve neighborhood safety perception via urban environment design programs.

More research is desired in this area. Using massive street view data, this study evaluates the street-view greenery in Guangzhou neighborhoods via a deep learning technique and examines the relationships between street-view greenery and fear of (neighborhood) crime in the Chinese setting under the guidance of environmental criminology theories.

### 1.1. Determinants of the Fear of Crime

Fear of crime is a criminological concept and its definition is not uniform. A large amount of literature was devoted to the conceptualization and operationalization of fear of crime [13,14,15]. Often, fear of crime is considered as a multidimensional concept, with three dimensions: affect, cognition, and behavior. The affective component refers to concerns and worries about encountering criminal incidents; the cognitive component reflects the perceived risk of victimization; the behavioral dimension refers to restrictions of action or defensive behaviors. In empirical studies, however, abundant criminological studies only use the affective fear of crime. Most studies use multiple indicators to measure fear of crime [16,17], while a few use one or two indicators [18,19].

A theoretical perspective of fear of crime is the vulnerability model [20]. According to this model, those physically or socially vulnerable people are expected to report higher levels of fear of crime. Women and the elderly are typically regarded as physically vulnerable populations; meanwhile, those with a lower level of personal income and/or education are considered as socially vulnerable populations. Research has demonstrated that the groups of women, the elderly, the lower-income population, and the less educated tend to report higher levels of fear [21,22,23]. The second influential theory is the disorder model which directly focuses on community characteristics. A neighborhood with physical deterioration (e.g., damaged public facilities) may be a reflection of the lack of social control, thereby causing fear of crime [24,25]. The third model, known as the social integration model, focuses on social integration, which is defined as people’s sense of belonging to their local community as well as their attachment to the neighborhood [26]. The social integration model posits that more socially integrated people tend to have a lower level of vulnerability and less fear of crime [27].

These findings are critical and can provide policy recommendations and practical program guidance. In addition, the physical characteristics of urban neighborhoods have also attracted the attention of researchers and policymakers.

### 1.2. Urban Greenery and Fear of Crime: Evidence

Due to the closed space environment, green spaces contribute to higher levels of fear in several studies [2,28,29]. Some research uses images to test the relationship between green space and fear of crime [29,30]. Some have explored this relationship by using questionnaires or surveys in the actual settings. Mak and Jim [1] reported that people were afraid of becoming a victim of crime when visiting the parks in Hong Kong. Meanwhile, several studies have found that people’s fear of crime depends on the visibility of green space. Woodland vegetation with good visual accessibility is beneficial to people’s perception of safety [4,30].

In many of the above studies, fear is a universal feeling (not crime-specific fear), or a general situational fear (it will disappear soon after leaving that environment) [31], or a stable fear of crime [31,32]. Regarding the stable fear of crime in the neighborhood, Maas and her colleagues [6] illustrated the relationship between the amount of green space and perception of neighborhood safety with a case in the Netherlands. Their findings suggested that residents living in a greener surrounding environment reported increased feelings of social safety. Mouratidis [7] verified the relationship between urban tree cover and increased perceived safety in 45 neighborhoods of the Oslo metropolitan area.

Some circumstantial evidence also suggests a possible relationship between green space and fear of neighborhood crime. A series of research has demonstrated that more residential vegetation can reduce crime rates [33,34,35]. Moreover, studies have examined the relationship between vegetation and incivilities [36,37]. For example, fewer problems are reported in neighborhoods with higher levels of maintained gardening. Additional evidence from studies that examine the relationship between residential vegetation and residents’ levels of aggression, violence, physical and mental health [38]. The majority have illustrated the positive effect of green space on residents’ wellbeing and physical health [9,39]. The higher the feeling of wellbeing and the healthier the body, the less likely the person will become a vulnerable individual, and thus might a lower fear of crime.

In sum, more residential vegetation has been linked with a lower crime rate, fewer incivilities, and less aggressive and violent behavior. There is a variety of evidence suggesting that more vegetation may be linked to a lower fear of crime in residential neighborhoods. But empirical evidence is still lacking in the street-view greenery field, as well as in non-Western contexts. Since street-view greenery in highly urban areas is usually well-maintained vegetation, street-view greenery may be associated with a lower fear of neighborhood crime.

### 1.3. Urban Greenery and Fear of Crime: Mechanism

From a criminological perspective, surveillance is an inhibitor of criminal activities. One of the earliest and most influential statements is *Jacobs’s Death and Life of Great American Cities* [40]. Jacobs [40] suggested that the presence of more ‘eyes on the street’ would deter criminal activities. In urban areas, it has shown that outdoor spaces with more trees are consistently greater used by residents than spaces without trees [41,42]. The more trees in an area, the greater the ambient population in that area. Offenders avoid areas with a greater likelihood of surveillance and intervention because their activities might easily be observed in these areas. Thus, a greater amount of vegetation might increase surveillance. Furthermore, Newman [43] proposed the ‘defensible space’ and suggested that criminals might be deterred by environmental signs, even if no one is present. This may be a function of territorial marking, as Kuo and Sullivan [3] proposed that well-maintained vegetation may constitute a particular territorial marker/sign. Therefore, implied surveillance can also prevent crime, and possibly the fear of crime.

However, how green space influences fear of neighborhood crime is rarely discussed via quantitative methods. Recently, some studies have demonstrated the relationship between green space and perceived neighborhood conditions, like social integration [44]. Perceived neighborhood conditions are important correlates influencing fear of crime. Further exploration of the link between green space and fear of crime, including the effects of mediation, is much desired.

Mediation means that a mediator mediates the relationship between the independent variable and the dependent variable. The mediator explains why this relationship exists. The relationship between the micro-environment and fear of crime in the neighborhood applies in two ways. First, direct signs of crime can indicate the possibility of crime. Second, environmental clues might arouse fear of crime, and the environmental clues symbolize the neighborhood’s ability to exercise informal social control. Collective efficacy and neighborhood incivilities may be driven by neighborhood structural characteristics, such as neighborhood poverty level, ethnic diversity, and crime rates. Brunton-Smith, Jackson, and Sutherland [45] suggested that collective efficacy and neighborhood incivilities are the bridges by which neighborhood structure characteristics affect fear of violence. Therefore, on one hand, urban street-view greenery might provide opportunities for neighborhood interaction, and contribute to social integration and attachment [44]. Meanwhile, social integration is a typical predictor of inhibiting fear of crime, so green space in neighborhoods might reduce the level of fear among residents. On the other hand, a higher level of street-view greenery heralds a more orderly and low-incivility neighborhood, and thus might lead to a lower level of fear of crime.

Therefore, we propose that street-view greenery may reduce fear of crime in the neighborhood in the following ways: by increasing informal and implied surveillance of the neighborhood, and by increasing the probability of intervention. Moreover, perceived conditions (such as neighborhood incivilities and social integration) might mediate the relationship between the street-view green space and fear of crime.

### 1.4. The Present Study

This study aims to analyze the associations and mechanisms between street-view greenery and fear of crime in the neighborhood among residents by using street-view greenery extracted from massive street-view pictures in central Guangzhou, China. The following are the research questions:

1. Is a higher level of street-view greenery associated with a lower fear of crime in the neighborhood?
2. What are the potential mediating roles of perceived conditions (physical and social incivilities, and social integration) on the relationship between street-view greenery and fear of crime in the neighborhood?

## 2. Materials and Methods

### 2.1. Description of the Study Area

**Figure 1:**
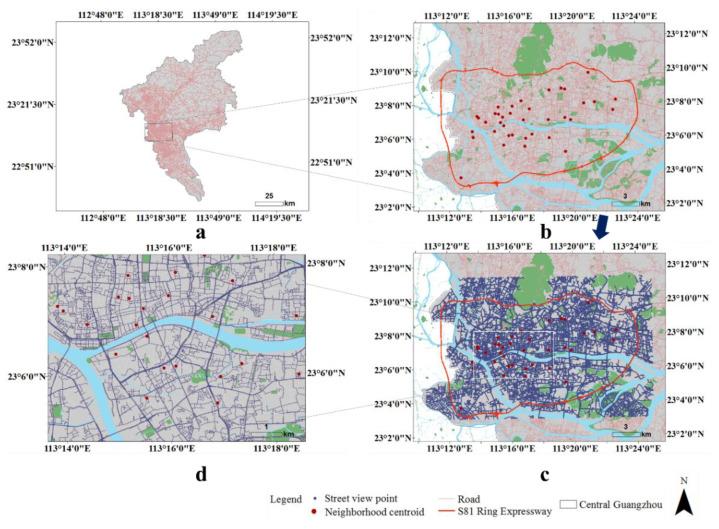
Distribution of sampled points for capturing street greenery images. (**a**) Guangzhou map; (**b**) central Guangzhou; (**c**) 73,289 street-view sample points with purple color; (**d**) a magnified area with street-view sample points).

Guangzhou (112°57’ E–114°30’ E; 22°26’ N–23°56’ N) is located in southern China, adjacent to the South China Sea. It is the capital and largest city of Guangdong province and the third-largest city in China, with an estimated population of 15 million in 2019. Guangzhou contains 11 administrative districts over an area of 7434 km^2^. As the area within the S81 Guangzhou Ring Expressway is the central and most urbanized area of Guangzhou, and the street view data is fully covered and is a highly representative area (in the suburbs of the city, the road network is less dense and the street view is not fully covered), we chose this area (central Guangzhou) as the sample area. This area covers an area of 380 km^2^ (Figure 1a).

### 2.2. Data

#### 2.2.1. Fear of Crime Data

The current study employs data from the Project on Public Safety in Guangzhou Neighborhoods (PPSGN), an interdisciplinary study aimed at understanding how neighborhood contexts affect the safety perception of residents in Guangzhou, China. The survey was designed by the research team and was distributed from January to April 2016 by the HOUSONWELL market research (http://www.hswell.com/), a professional and well-known market research company in China. The survey process is as follows. First, the research team and the market research company trained the questionnaire interviewers. Then, the interviewers entered the neighborhoods, randomly selected participants (participants should be residents of the neighborhood), conducted the questionnaire face-to-face, and recorded the answers on the tablet computer. The respondents were interviewed at the door of their residence or in the open space within their apartments. All participants were given some gifts (tissues and pens) as rewards. For more details about the survey collection, see Jing et al. [22].

Since college students and people under the age of 18 are greatly affected by the campus environment, they were politely asked not to participate in the questionnaires. Using a probability sample procedure, the PPSGN collected information from 1994 Guangzhou residents in 91 neighborhoods, with a response rate of 87.4%. As the case area in this study was central Guangzhou, finally, 773 participants from 37 neighborhoods (within central Guangzhou) in the total sample were selected (Figure 1b). We selected approximately 20 participants from each neighborhood (Mean = 20.89, Min = 18, and Max = 29).

#### 2.2.2. Street-View Greenery Data

**Figure 2:**
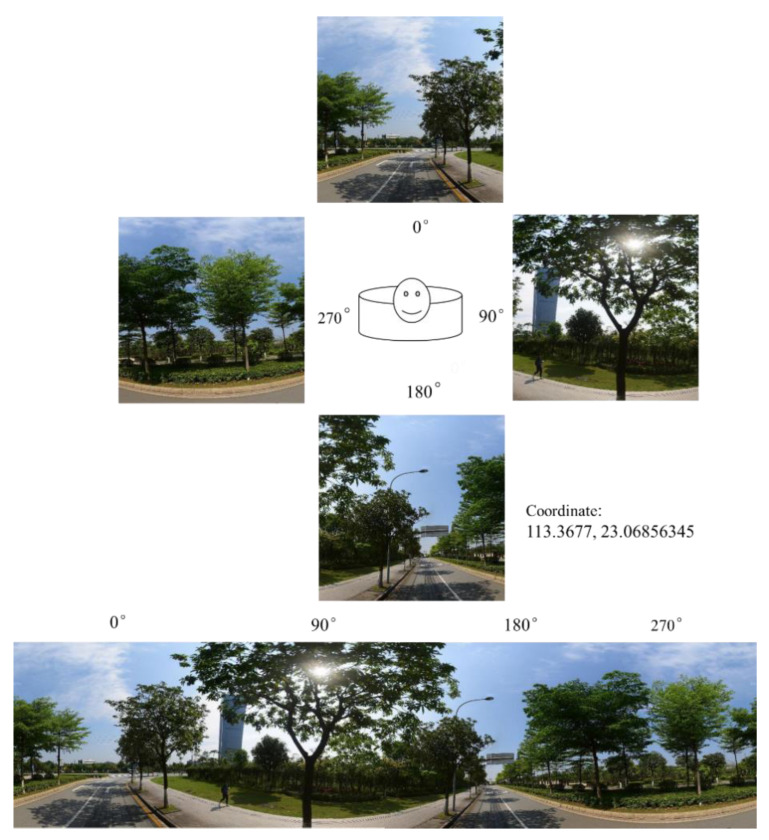
Street-view greenery images capturing an example area.

We evaluated green space per neighborhood based on a series of street view images collected in December 2018. The images were crawled from Baidu Map, the Chinese equivalent of Google Maps (Google products cannot be accessed in mainland China). It is China’s leading map services provider, has the largest image coverage, and provides street view pictures taken from various positions. Several studies involving Chinese cities have used Baidu Map data [46,47]. Based on the central Guangzhou road map, we constructed 74,802 points along with the road network. The sampled points are 50 m apart, which is a compromise between the level of detail and costs. According to these locations, the closest pictures in the horizontal direction were queried through a URL link and crawled through the API (application programming interface). To include the entire streetscape at each sampled point, we acquired images taken in the four directions (i.e., 0, 90, 180, and 270 degrees) (Figure 2). The size of each image is 512 × 512 pixels with a vertical angle of 0 degrees (pitch = 0). In total, through the Python programming language, 73,289 street view images with four directions in central Guangzhou were obtained (Figure 1c,d).

### 2.3. Measures

#### 2.3.1. Self-Reported Fear of Crime Indicator

Fear of crime. Fear of crime was assessed using a question with five items from the PPSGN survey. (1) How fearful are you of being robbed in the neighborhood? (2) How fearful are you of being harassed in the neighborhood? (3) How fearful are you about your home being broken into? (4) How fearful are you about your money or property being stolen in another way? (5) How fearful are you about being beaten or hurt when you walk alone at night in the neighborhood? Respondents were asked to indicate a response to these questions on a 5-point Likert-type scale, where 1 stands for ‘not fearful’, to 5 standing for ‘very fearful’. Fear of crime is the average of the five items, with higher values indicating greater fear. The internal consistency of the measure is confirmed by a high value of 0.85 on Cronbach’s α. α has the same meaning in the following related descriptions.

#### 2.3.2. Individual-Level Variables

Demographic Information. In general, females and old people might have relatively high levels of fear, so gender (female = 1) and age (continuous variable) were assessed. This study also measured personal income (ordinal variable, ranging from 1 to 7, meaning from low to high) and the education level (ordinal variable, ranging from 1 to 8, meaning from low to high). The measure of prior victimization was to ask whether the respondent had previously been victimized in the last three years (1 = yes).

Perceived physical and social incivilities. Respondents were asked about a series of conditions separately and whether it was present in their surroundings. Perceptions of neighborhood incivilities are composite variables (summed across several variables). The variable perceived physical incivilities reflects a scale ranging from 0 to 5, including the following four items: abandoned cars and/or trash, damaged public facilities and/or poor lighting, graffiti and/or disordered advertisements, and noisy neighborhood environment (α = 0.82). The variable perceived social incivilities reflects an increasing scale ranging from 0 to 5, including the following four items: drunken persons on the streets, teenagers gathering on the streets, suspicious strangers, and residents conflicting on the streets (α = 0.84).

Social integration. Respondents were asked four items about their perception of social integration in their neighborhood. This variable reflects a scale ranging from 0 to 5, comprised of visiting neighbors informally, chatting with neighbors, borrowing things like tools from neighbors, and belonging to a network in which neighbors help each other (α = 0.77).

#### 2.3.3. Neighborhood-Level Variables

Neighborhood street-view greenery. Based on the boundary of the census neighborhoods, we calculated the street-view greenery at the neighborhood-level. Census neighborhoods, the smallest administrative units, are derived from the 2010 Census (The 2010 Census refers to the 2010 National Census, which is the latest national census data in China so far). The equation of the neighborhood street-view greenery is as below:

(1) $$SVG=\frac{\sum_{1}^{n}X_{n}}{n}.$$

In Equation (1), X_1_, X_2_, X_3_, …, X*_n_* refer to the street-view image points within a neighborhood area, *n* means the number of images within a neighborhood, and *SVG* refers to the average street-view greenery value of this neighborhood.

When investigating the relationship between fear of crime and the visibility of green space in a person’s living environment, other neighborhood-level factors that could influence fear of crime ought to be controlled. People tend to feel less safe in neighborhoods with higher concentrated disadvantages [23], such as areas with high crime rates [48]. In this study, we controlled for the population density, the percentage of migrant population, and the crime rate in the census neighborhood area.

Population density. Population density refers to the number of people in the neighborhood per square kilometer.

The proportion of migrant population. Migrant population percentage was calculated as the ratio between the number of non-local hukou population (migrants from outside Guangzhou) and the number of neighborhood inhabitants.

Crime rate. The number of robbery and burglary incidences per 100,000 of the population per year during the period of 2014 and 2015 was calculated for each of the 37 neighborhoods. Crime data was obtained from Guangzhou Public Security Bureau.

### 2.4. Deep Learning for Image Segmentation

Yang et al. [49] proposed a Green View Index (GVI) to evaluate the visibility of urban greenery, which was defined as the ratio of the total green area of four pictures taken at a street intersection to the total area of the four pictures. Then, Li et al. [50] assessed the street-view greenery by pixel-wise classification based on massive google street view imagery. However, there are some limitations in pixel-wise classification using additive colors of the picture, such as the indistinguishable natural and artificial green objects. To overcome the limitation, the present study implemented a deep learning approach to extract street-view greenery from massive street images.

**Figure 3:**
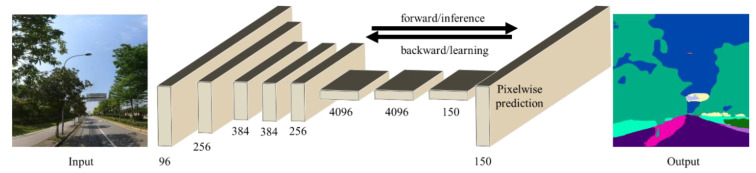
Street view image segmentation by a fully convolutional network (FCN-8s).

We applied a semantic segmentation technique called FCN-8s (full convolutional network for semantic segmentation) to identify green space from street view image data [11,51]. The FCN-8s was trained based on the ADE_20K dataset, which had a total of 20,210 training data and 2000 validation data. To train the FCN-8s, the scan window was set to 500 × 500 pixels, and the learning rate was set to 0.1, and the early-stopping minimum learning rate was set to 0.001. The batch size was set to 32. Two sets of Nvidia GTX 1080ti graphics processors were used to train the FCN-8s model. The trained FCN-8s showed good performance for semantic segmentation. The result of a pixel-by-pixel comparison shows that, in the experiment using the ADE_20k dataset, the accuracy of the trained FCN-8s on the training dataset is 0.81 and the accuracy on the test dataset is 0.67 (an accuracy of more than 0.60 is considered to be a good performance in pixel-by-pixel semantic segmentation). For more technical details of the trained FCN-8s model, see the work of Yao and his colleagues [52], while a series of research has notably used the trained FCN-8s to assess streetscapes in Chinese cities with desirable results [11,53]. After the image segmentation, this approach identified 150 elements, including common ground objects such as trees, sky, persons, cars, roads, and buildings, and the proportion of green space (including trees, grass, and plants) was calculated (Figure 3).

In each sampling point, streetscape green space represents the ratio of the number of green space pixels per image summed over the four directions to the total number of pixels per image summed over the four directions. Then, the data of points were aggregated to the neighborhood level.

### 2.5. Analytical Strategy

As the dataset shows a nested structure, this study evaluated the associations between street-view greenness and fear of crime through multilevel regression modeling, while controlling several individual and neighborhood variables. Numerous studies on fear of crime have illustrated the satisfactory performance of multilevel modeling [6,25]. The multilevel analysis was performed with HLM 6.08 [54] in this present study. Here, the models were expressed as follows:

(2) $$Fear\;of\;crime=\;\beta_{0}+\;\sum_{q}\beta_{q}X_{qi}+\;r_{i}$$

$$\beta_{0}=\gamma_{00}+\;\sum_{s}\gamma_{s}W_{s}+\mu_{0}$$

$$\beta_{q}\left(Mean\;X_{qi}\right)=\gamma_{q0}+\mu_{q}.$$

In Equation (2), $\beta_{0}$ was the intercept; $\beta_{q}$ was the slope of the factor *q* on the fear of crime; $X_{qi}$ was the factor *q* for individual *i*; $r_{i}$ was the error term that was represented to the random effects. Where $\beta_{0}$ was modeled as a function of neighborhood contextual characteristics $W_{s}$ (neighborhood street-view greenery, etc.); $W_{s}$ was the factor *s* for its neighborhood; the models were specified with a random error coefficient $\;\mu_{q}$. This allowed the intercept to vary as a function of neighborhood features and any unique neighborhood effects.

First of all, the present study estimated a one-way analysis of variance with random effects, to explore how much influence was produced by the neighborhood-level factors. Consistent with Wyant [16], the intraclass correlation coefficient indicated that a 15.19% variation of the fear of crime can be explained by neighborhood-level variables, and the rest was attributed to individual-level change and random error. Then, two multilevel models were conducted separately. In a multilevel regression model, a variable with no meaningful zero value needs to be centered. Therefore, all individual-level variables were mean grouped, and all neighborhood-level variables were grand mean centered in the current study.

Mediation analysis was undertaken in four steps following Baron and Kenny [55] and previous research [56]. Conditions for mediation are that the predictor variable (street-view greenery) must influence the mediator; the mediator must influence the outcome variable (fear of crime); the association between the predictor and outcome is eliminated or weakened when the mediator is included in the model. The mediating role of the perceived condition on the relationship between street-view greenery and fear of crime was assessed by conducting three separate statistical models. First, the correlations were calculated between the possible mediators (physical and social incivilities, and social integration) and fear of crime. Possible mediation was only considered for combinations with statistically significant correlations. In these cases, the second step was to compute a linear regression where the possible mediator was entered as the dependent variable and street-view greenery was taken as the independent variable. The last step was to perform a multiple regression using a saturation model, in which both the mediator and street-view greenery were entered as independent variables, and the fear of crime was input as the dependent variable. A total of 5000 bootstrap samples were used to examine the statistical significance of the mediating variable. This method achieves 95% CI (confidence intervals) of the indirect effect. A bootstrapped 95% CI not straddling 0 is considered statistically significant [57]. Mediation analysis was conducted using the PROCESS v3.5 for SPSS Statistics 22 (Hayes [57]).

## 3. Results

### 3.1. Descriptive Statistics

**Figure 4:**
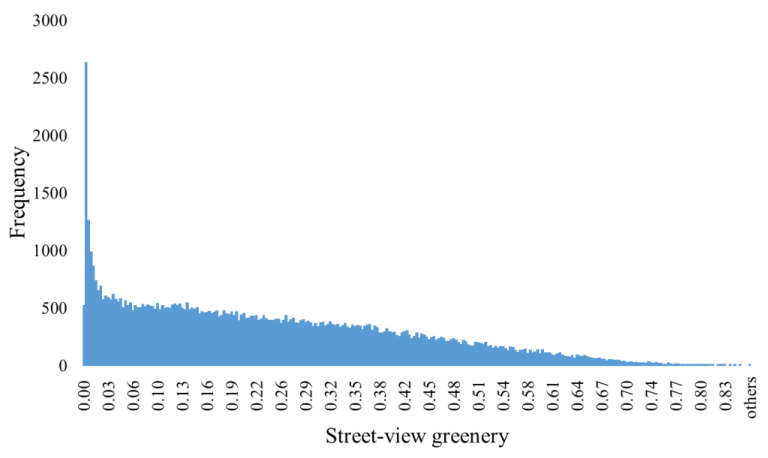
Histogram distribution of street-view greenery.

**Figure 5:**
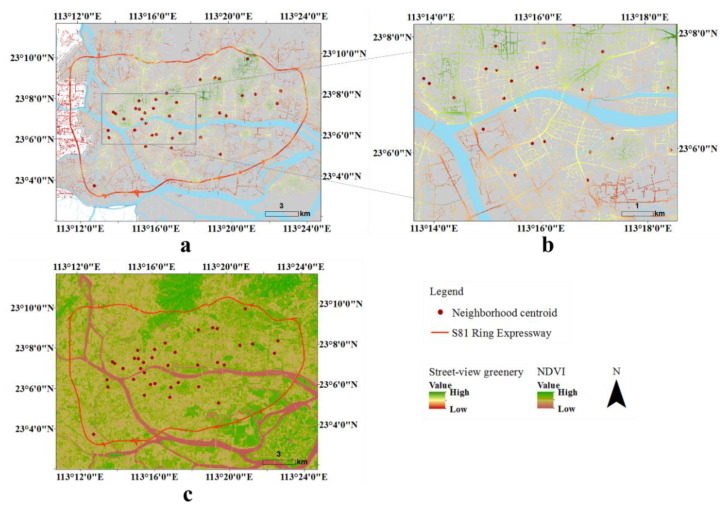
Visualization of green space in central Guangzhou (**a**) greenery derived by the street-view imagery; (**b**) a zoomed-in view; (**c**) NDVI derived by satellite imagery on 7 February 2016).

Among the 73,289 image sites in central Guangzhou that were finally chosen, street-view greenery scores vary from 0 to 0.86, with a mean value of 0.234. The histogram distribution of street-view greenness for all image sites in Guangzhou is shown in Figure 4. Compared with the vegetation greenness derived by satellite imagery (NDVI, the normalized difference vegetation index), the visualization of greenness in the street shows that spatial characteristics that were broadly similar but different in detail (Figure 5a,c). Converged to a planar spatial scale, the mean of neighborhood-level street-view greenery is 0.27.

The descriptive statistics of participants’ individual and neighborhood characteristics were presented in Table 1. The sample was composed of 49.1% women, with an average age of 40.42 years. The average fear of participants was not high (Mean = 2.34). We also conducted the correlation analysis for the individual and neighborhood characteristics separately. No problems with colinearity were detected.

### 3.2. Multilevel Results

In the individual-level Model (Model 1, Table 2), women were more fearful than men and perceived physical and social incivilities were positively correlated with fear of crime. Respondents who perceived higher physical or social incivilities in the neighborhood had a higher fear level. Higher perceived social integration was associated with a lower fear of crime. At the individual level, we found no evidence that age, levels of education, personal income, and prior victimization were significantly related to fear of crime.

The neighborhood-level model (Model 2, Table 2) shows the main effect of street-view greenery. In line with expectations, after controlling relevant variables, neighborhood street-view greenery was significantly related to fear of crime (coeff. = −1.01, *p* < 0.05); thus, respondents exposed to more street-view greenery in the living environment reported lower levels of fear than respondents exposed to less street-view greenery in the living environment. Meanwhile, unexpectedly, the migrant population was statistically associated with a lower fear of crime.

### 3.3. Mediated Results

We conducted a series of mediation effect analysis to test whether perceived conditions (physical and social incivilities, social integration) mediate the relationship between street-view greenery and fear of crime. A mediation analysis PROCESS v3.5 for SPSS was performed.

**Figure 6:**
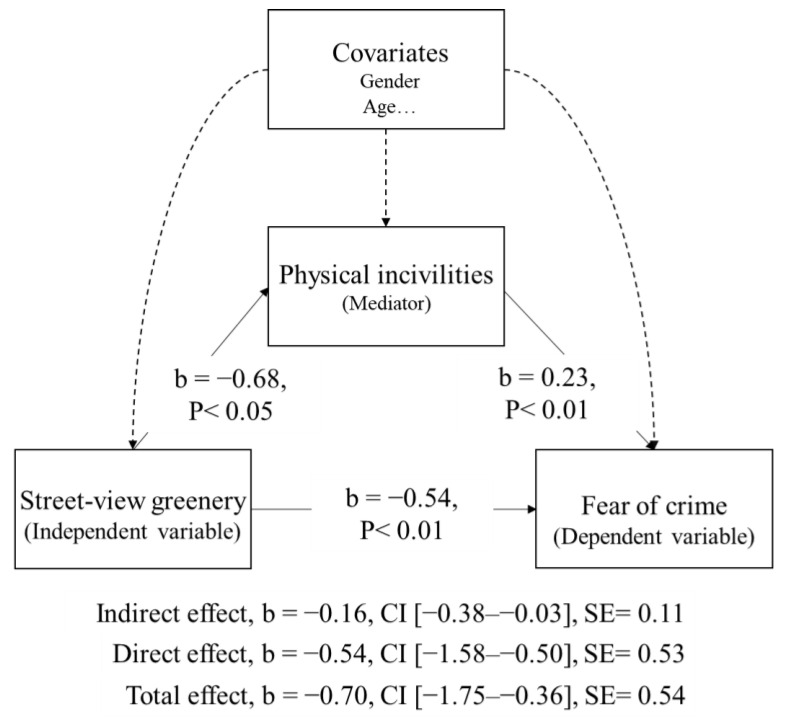
Mediation model of street-view greenery as a predictor of fear of crime, mediated by physical incivilities (b is an unstandardized regression coefficient, and CI is the confidence interval for the bootstrap approaches between BootLLCI and BootULCI).

Demographic and socioeconomic factors were included as covariates for models presented in this study, as they showed associations with certain variables of the present study. The mediation results of physical incivilities showed (see Figure 6) that: (1) street-view greenery predicted perceived physical incivilities (b = −0.68, *p* < 0.05); (2) physical incivilities predicted fear of crime (b = 0.23, *p* < 0.01); and (3) street-view greenery predicted fear of crime (the direct effect, b = −0.54, *p* < 0.01). It was also reported that street-view greenery as a predictor of fear of crime was mediated by physical incivilities (the indirect effect, b = −0.16, CI (confidence interval) [−0.38–−0.03], SE = 0.11). The mediation test was performed by calculating CI for the ‘indirect effect’ using bootstrap methods. For the size of the effect, all CI did not contain zero point estimates; thus, 0 can be excluded as a probable value for the direct and indirect effects.

When other perceived conditions (perceived social incivilities and social integration) were included as the mediator for models separately, the mediation effect was not significant. Thus, no empirical evidence demonstrates the effect of street-view greenery on fear of crime via social incivilities or social integration.

## 4. Discussion and Conclusions

This study is the first to examine the linkage between fear of crime in the neighborhood and exposure to green space at the street level among residents in China. Although remote sensing-based metrics of green space are widespread in environmental studies, we take an alternative avenue to assess green space, relying on deep learning techniques and street view data. The results lead us to conclude that in central Guangzhou, a high level of street-view greenery in people’s living environment is generally associated with a lower fear of neighborhood crime. This relationship is concurrent with the positive relationship between green space and people’s health and previous research. The mediation analyses show that perceived physical incivilities partially mediates the relationship between street-view greenery and fear of crime. It makes a further understanding of the relationship between green space and fear of crime and guides that the safety improvement programs need to consider both the physical design and social factors.

More importantly, a traditional view holds that green space itself is not associated with neighborhood safety perception. Residents in neighborhoods with more green space feel safer because these neighborhoods may be more affluent. The finding here shows that a higher level of street-view greenery in highly urbanized areas is systematically and independently associated with a lower fear of neighborhood crime, after controlling for other individual and neighborhood characteristics.

### 4.1. Contributions to the Understanding of Green Space and Fear of Crime

One contribution of this work is to further illustrate an environmental criminological perspective, that is, more urban green space is associated with higher perceived neighborhood safety. Greener streets receive greater use, thereby increasing informal surveillance. Also, many forms of street vegetation preserve visibility and therefore it does not promote fear of crime in the neighborhood. This finding suggests that fear of crime prevention concerns does not justify removing street vegetation in highly urbanized areas.

Although this is the first study to demonstrate such a link, the findings are consistent with previous work linking vegetation with incivilities [36], perceived social safety [6], and crime rates [3]. Consistent with the findings of Maas et al. [6] and Mouratidis [7], we found that safety perception is associated with green space. The results obtained here are based on street-view greenery and neighborhood perception of safety, whereas previous literature is based on satellite imagery and social safety perception.

Based on the deep learning approach, some scholars [50,58] collected perceived safety data and did some research on the relationship between perceived safety and environmental characteristics. The principle of street-view safety perception scores is to show participants two geotagged images and ask them to choose the image that looks safer. Then, any picture can obtain a safety perception score by using the trained model based on the crowdsourced data. Li, Zhang, and Li [50] found that the visibility of green vegetation plays an important role in enhancing perceived streetscape safety. Unlike the perceived safety provided by deep learning, in our study, fear of crime refers to the respondents’ perception of safety in the neighborhood where they live. Even so, the work of Li and his colleagues indicated that green space contributes to enhanced safety perception of images. It provides indirect support for explaining the association between street green space and fear of crime.

Another contribution of the work here was to help resolve a previous puzzle on residential vegetation and feelings of safety. It is generally believed that objective or perceived green space is related to fear of crime because densely-vegetated urban neighborhoods represent lower levels of poverty and incivilities/disorder. However, this study demonstrates that neighborhood physical incivilities/disorder partially but not completely mediates the relationship between street-view greenery and fear of crime. Thus, in essence, street-view greenery may have direct and indirect effects on fear of crime.

An unexpected finding is that neighborhood migrant percentage is related to lower levels of fear of crime. Although neighborhoods with a higher percentage of migrants or minorities are associated with fear of crime in the West [59,60], the role of neighborhood migrants may be controversial in China. Using a variable of rural migrant concentration, Liu et al. [61] did not find a significant relationship between neighborhood migrants and fear of crime. Hence, two reasons may explain this unexpected finding. The first one may be the measure of neighborhood migrants. In the present study, migrants refer to general domestic migrants who have lived in the neighborhood for more than half a year, not foreign immigrants, nor just rural migrants. Also, even if the places inhabited by rural migrants are considered to be poor, the corresponding census neighborhood area is larger. Therefore, even if a census neighborhood has a high proportion of migrants, this neighborhood may not be poor. Second, the study area of this study is in central Guangzhou. A higher percentage of migrants might represent a higher economic level, as those areas with good economic conditions attract migrants to settle. It is necessary to examine this finding further in future research.

As this study used the 50 m granularity and neighborhood-level street-view greenery, the MAUP (Modifiable Areal Unit Problem) may arise [62]. The street view greenery with different granularities and units may lead to bias in the results. Therefore, this study reminds the existence of the MAUP and expects that future research will minimize this problem when exploring the relationship between green space and fear of crime.

### 4.2. Practice Implications and Further Research Directions

As street vegetation may inhibit fear in urban neighborhoods, it seems appropriate to increase street greenery in crowded inner-city areas to create neighborhoods with a higher safety perception. If there is more evidence, then a series of street greenery strategies are recommended. Trees and grassland along the street can be planted more, because they provide good visibility. Then, green space strategies can continuously maintain the street green space to avoid the negative impact of green space on fear of crime. Finally, since green space can be used as a sign of territory, planting green space in main road nodes may promote a greater beneficial effect.

The findings also have implications in other areas. Results show that fear of crime is simultaneously influenced by the physical environment and social factors. Ultimately, the largest reductions in fear of crime will come from strategies that address social and cultural factors, such as reducing intense poverty, improving the level of collective efficacy, and reducing the level of incivilities in the neighborhood. These strategies can help play a positive role in physical designs such as planting street greenery affecting safety perceptions.

Our findings set the stage for further exploration of the relationship between urban street vegetation and fear of crime. With the development of deep learning techniques, the results of image segmentation are constantly enriched. Future studies might use a deep learning approach to detect the maintenance level and specific characteristics of vegetation (e.g., height, living vegetation volume, and vegetation health) in the street scenes in large quantities, and examine the fear of neighborhood crime associated with these factors. More detailed street-view greenery can examine more sophisticated relationships.

Street view images and machine learning are powerful tools for environmental exposure assessments in urban landscapes. Companies such as Baidu Maps and Google Maps provide geo-referenced and publicly available street view image databases with broad spatial coverage, and the development of novel location-based environmental exposure measures (e.g., sky view index, street-view water space, etc.) to explore environment–crime relations is meaningful.

Our mediation models only demonstrated that physical incivilities partially explained the impact of street-view green space on fear of crime. Future research can examine more potential mediators, such as perceived neighborhood attachment, informal social control, and physical activity. Meanwhile, social control/integration may also result in more green plants being planted in neighborhoods. The mechanism of green space on fear of crime needs more explanation in future research. In addition, the effect of green space on fear of crime might vary across types of neighborhoods. The next step should compare the effect of street green space on fear of crime in different types of neighborhoods. Finally, even if street green space reduces the fear of crime, is this relationship linear or curvilinear? Living in an area with very high green space exposure, the benefits of greenery may be reduced because of the desensitization effect. Future research might also focus on the possible nonlinear relationship between green space and fear of crime.

### 4.3. Limitation

The present research has a limited ability to make causal inferences regarding our evaluated associations. The first limitation is the temporal and spatial resolution of street view data. We did not obtain the street view during the same period of the questionnaire survey because of the technological restriction of Baidu street view rules. Street view is not updated every year, which does not accurately reflect the changes in the street environment. Besides, there are some small roads or alleys that people often walk, but street view cars cannot enter to capture images. The spatial distribution of street view is not even in some areas. Secondly, our data do not provide any information on the actual use of green space by the respondents, such as the frequency of visiting green space and time during the green space. This limits the data’s ability to reveal the relationships between objective street green space, perceived street green space, and fear of crime. Thirdly, crime rates are aggregated at the neighborhood level, but in fact crime is highly concentrated in hot spots. Hence, even in high-crime neighborhoods, most places are safe and only a few are dangerous. Fourthly, more factors still need to be considered in the relationship between urban greenery and fear of crime, such as the difference between day and night, and different types of vegetation. Lastly, a major limitation of our analysis is that it is observational. There are likely added, unaccounted for differences between the locations that are not accounted for in the model. Although several limitations still need to be overcome, the present study demonstrates the relationship between a novel street-view greenery and fear of crime.

## Figures and Tables

**Table 1:** The characteristics of the study variables.

	Mean	SD	Min	Max		% (N)
Individual level						
Age	40.21	14.68	19	84		
Gender (female = 1)	0.49	0.50	0	1	Female	49.3% (381)
					Male	50.7% (392)
Level of education	4.46	1.64	1	8		
Personal income	3.67	1.24	1	7		
Prior victimization experience (yes = 1)	0.35	0.48	0	1	Victimization	35.2% (272)
					Non-victimization	64.8% (501)
Perceived physical incivilities	2.82	0.83	1	5		
Perceived social incivilities	1.85	0.76	1	5		
Perceived social integration	3.42	0.83	1	5		
Neighborhood level						
Street-view greenery	0.27	0.07	0.12	0.44		
Population density (per km^2^)	43,827	27,063	3501	105,271		
Percentage of migrant population	0.17	0.13	0.02	0.65		
Crime rate (per 100,000 people)	1330.27	1762.50	183.87	8446.42		
Dependent variable						
Fear of crime	2.44	0.99	1	5		

**Table 2:** Multilevel results of the effect of street-view greenery on fear of crime (with robust standard errors).

	Model 1			Model 2		
	Coeff.	SE	*p*-Value	Coeff.	SE	*p*-Value
Intercept	2.44 **	0.07	0.00	2.44 **	0.07	0.00
Level 1—Between individuals						
Age	−0.00	0.00	0.18	−0.00	0.00	0.18
Gender (female = 1)	0.20 **	0.07	0.00	0.21 **	0.07	0.00
Level of education	0.03	0.03	0.33	0.02	0.03	0.36
Personal income	0.05	0.03	0.05	0.05	0.03	0.05
Prior victimization experience (yes = 1)	0.10	0.07	0.15	0.10	0.07	0.18
Perceived social integration	−0.10 **	0.05	0.00	−0.10 *	0.05	0.02
Perceived physical incivilities	0.14 *	0.06	0.02	0.15 *	0.06	0.01
Perceived social incivilities	0.23 **	0.06	0.00	0.23 **	0.06	0.00
Level 2—Between communities						
Street-view greenery				−1.01 *	0.87	0.02
Neighborhood population density				0.00	0.00	0.25
Percentage of migrant population				−1.01 *	0.39	0.03
Crime rate (per 100,000 people)				0.00	0.00	0.32
Variance components	Var.	*χ^2^*	*p*	Var.	*χ^2^*	*p*
Between individuals	0.64	-	-	0.64	-	-
$\mathrm{Between}\;\mathrm{neighborhoods}\;\beta_{0}$	0.16	221.38	0.00	0.15	191.16	0.00

Note: * *p* < 0.05, ** *p* < 0.01.

## Data Availability

Data of the street-view greenery presented in this study are available on request from the first author. Data of the PPSGN survey are not publicly available due to data protection regulation. The 2010 census data are openly available on request from the National Bureau of Statistics of P.R. China (http://www.stats.gov.cn/).
